# Factors related to NT-proBNP levels in HIV patients aged over 40 years

**DOI:** 10.1186/s12981-015-0058-7

**Published:** 2015-05-10

**Authors:** Julián Olalla, Elena Crespo, Javier De la Torre, Marco Sempere, Alfonso Del Arco, José Luis Prada, Rocío Malvarez, Javier Pérez, Javier García-Alegría

**Affiliations:** Internal Medicine Unit, Costa del Sol Hospital., 29603 Marbella, Spain; Clinical Analysis Laboratory, Costa del Sol Hospital, Marbella, Spain

**Keywords:** Hiv, Aids, Cardiovascular risk, Nt- probnp, Highly active antiretroviral therapy

## Abstract

**Objective:**

To determine the levels of NT-pro BNP in HIV patients over 40 years who are receiving highly active antiretroviral therapy (HAART) and investigating potential independent clinical or laboratory factors.

**Method:**

We determine levels of NT-pro BNP in peripheral blood of HIV patients from Costa del Sol Hospital, over 40 years.

We collected epidemiological, classical cardiovascular risk factors and variables associated with HIV infection status. The qualitative variables were compared using the χ2 test. NT-proBNP levels were taken as the dependent variable. The association between these levels and the quantitative variables were studied by analysis of variance (ANOVA), and the association with the qualitative variables, using Student’s t test.

**Results:**

Nt-pro BNP levels were determined in 146 HIV patients. We assess the 10-year cardiovascular risk calculated by the Framingham equation, 59 (41.5%) were classified as low risk, 46 (32.4%) as a moderate risk and 37 (26.1%) as a high risk. The higher levels of NT-pro BNP were found in women, and in those patient with lower filtration rate and high levels of triglycerides. An association was also observed between higher levels of NT-proBNP and the recent use of lamivudine and fosamprenavir. After a multivariate analysis we found an association between higher levels of NT-proBNP and the current use of fosamprenavir and a lower glomerular filtration rate.

**Conclusions:**

We found, with the limitations of a small serie, that higher levels of NTproBNP in HIV patients could be linked to the occurrence of cardiovascular events, this fact suggest that NTpro BNP could be used in patients at moderate or high vascular risk in order to optimise the primary prevention of vascular events.

## Background

Older population cohorts in HIV patients have expanded considerably in recent years [1,2], so the infection is increasingly accompanied by age-associated comorbidities, including cardiovascular events (Ischaemic heart disease, stroke, heart failure or peripheral artery disease) [3].

These comorbidities are gaining increansing importance in mortality rates of patients infected with HIV [4]. According to several authors it seems clear that the incidence of cardiac ischaemic events is higher among patients with HIV infection compared to those who are uninfected, and even more in those who are receiving highly active antiretroviral therapy (HAART) [5]. On the other hand there is a study in which proves that CV events were higher among those who were not receiving HAART [6]. In part, this fact (the correlation of CV risk and treatment) is attributed to the use, whether recent or cumulative, of certain nucleoside analogues (abacavir, didanosine) and protease inhibitors (lopinavir, fosamprenavir (fAPV)).

Furthermore, it has recently been determined that the incidence of heart failure is almost twice as high among patients with HIV infection than in those uninfected patients, and it is comparable to traditional risk factors such as hypertension or diabetes [7]. Again, this increased incidence of heart failure has been linked to antiretroviral treatment, and specifically to the recent use of tenofovir (TDF) in one study [8]. Use of protease inhibitors has been related with another surrogate marker of vascular disease, the ankle-branch index [9].

B-type natriuretic peptide (BNP) is a polypeptide that is secreted by ventricular myocytes in response to pressure overload on the ventricular wall. Once BNP has been segregated, it divides into an active peptide and the N terminal fragment of BNP (NT-proBNP).

Studies have established the value of BNP and of NT-proBNP in the diagnosis and prognosis of ventricular dysfunction and in the prognosis of acute coronary syndromes [10-12]. It is considered a risk marker of vascular events in the general population [13] and also in patients with HIV infection [14].

However, few studies have focused on determining levels of NT-proBNP in patients with HIV infection, relating them to increased systolic pressure in the pulmonary artery [15] and the presence of comorbidities [16]. There is also some evidence that ART could be involved in increased left ventricular mass, and thus an analysis of these biomarkers could provide an additional prognostic measure for our patients [17].

In this paper, we explore the hypothesis that ART or one or more of its components might be associated with higher levels of NT-proBNP.

## Material and Methods

The patients were part of a previous research series [16] created to study the left ventricular mass in patients with HIV infection. The patients in this serie who were aged over 40 years were invited to cooperate in the present study, to determine levels of NT-proBNP in peripheral blood. When consent was given, a serum sample was extracted, frozen to −70°C and stored until needed for the NT-proBNP analysis.

NT-proBNP (amino acid residues 1–76) was measured using the Dimension RxL Max Analyzer (Siemens), lower limit of detection 10 ng/l. For all patients, epidemiological such as age and gender, and clinical (duration of HIV, duration of ART, BMI…) data were compiled, together with the classical cardiovascular risk factors and variables associated with HIV infection status.

These aspects are described in detail in the descriptive part of the series as a whole.

## Statistical analysis

Data were collected directly in SPSS 15.0 (Statistical Package for the Social Sciences, Inc. Chicago, Illinois, USA).

The quantitative variables were described using the mean with a 95% confidence interval (95% CI), and the qualitative variables were expressed as percentages.

The qualitative variables were compared using the χ2 test. NT-proBNP levels were taken as the dependent variable. The association between these levels and the quantitative variables were studied by analysis of variance (ANOVA), and the association with the qualitative variables, using Student’s t test, after verifying the normal distribution of the samples; when this condition was not fulfilled, the Mann–Whitney U test was applied.

The variables that presented a statistically significant association with NT-proBNP levels (p < 0.05) were included in the multivariate analysis, using a forward stepwise method. Statistical significance was set at p < 0.05.

## Results

### Characteristics of the participants

NT-proBNP levels were determined in 146 patients. The main characteristics of the participants in the study are shown in Table 1.

**Table 1:** **Main characteristics of the patients included in the study**

**Parameter**	
Male sex	111 (76%)
Age, years	50.14 (48.79-51.50)
Duration of HIV infection, years	10.61 (9.47-11.74)
Duration of ART, years	7.02 (6.16 - 7.88)
Naïve	16 (11%)
Nadir CD4 lymphocyte count	260.39 (222.01 - 298.77)
CD4 lymphocyte count at the time of the study	633.66 (581.99-685.33)
VIH-1 viral load <50 copies/mL	108 (74%)
Glomerular filtrate (mL/min)	105.03 (100.67-109.39)
Total cholesterol (mg/dL)	196.97 (189.82-204.13)
HDL cholesterol (mg/dL)	49.42 (46.78-52.06)
LDL cholesterol (mg/dL)	116.96 (110.92-122.99)
Triglycerides (mg/dL)	167.28 (149.43-185.12)
BMI	24.76 (24.05-25.46)
Systolic BP (mmHg)	129.32 (126.21-132.44)
Diastolic BP (mmHg)	77.65 (75.63-79.67)
10-year cardiovascular event risk (%)	14.81 (12.96-16.66)

The main route of acquisition of infection was the use of injected drugs (34.9%), followed by heterosexual contact (32.2%) and homosexual contact (27.4%).

A total of 56 patients had HCV coinfection (38.4%), and 24 (16.4%) had HBV coinfection.

With respect to the classical risk factors, 11 patients (7.5%) were diabetic, 21 (14.4%) had hypertension, 40 (27.4%) had dyslipidaemia and 78 (53.4%) were current smokers. Nine patients (8.2%) had prior vascular disease: of these, five had cerebrovascular disease, three had ischaemic heart disease and one had peripheral arterial disease. Seven patients had a family history of cardiovascular disease. Nine (6.2%) were receiving concomitant antiplatelet therapy, ten (6.8%) were being treated with beta-blockers, six (4.1%) with ACE inhibitors (angiotensin-converting-enzyme inhibitors), five (3.5%) with ARA-II (angiotensin II receptor antagonists), twenty (13.7%) with statins, ten (6.8%) with fibrates and five (3.4%) with metformin.

In regard to the 10-year cardiovascular risk calculated by the Framingham equation, 59 (41.5%) were classed as low risk, 46 (32.4%) moderate risk and 37 (26.1%) high risk, with an average risk of a vascular event within 10 years of 14.81%.

The levels of NT-proBNP (pg/mL) were observed to be higher among the women than in men (81.6 vs. 65.32, p = 0.002). Older age was also associated with higher levels of NT-proBNP (p = 0.005) but it was not related to duration of HIV or ART.

There was no difference between treatment-naive and experienced patients (28.19 vs. 74.27, p = 0.1). With respect to the analytical parameters, no relation was found with the nadir CD4 level or the level at the time of the study, nor with detectability. Levels of NT-proBNP were higher among the patients with a lower glomerular filtration rate (p = 0.028) and among those with higher levels of triglycerides (p = 0.005).

An association was also observed between higher levels of NT-proBNP and the recent use of Lamivudine (3TC) (51.55 vs. 108.89, p = 0.02) and fosamprenavir (52.82 vs. 223.83, p = 0.01) and between the recent use of TDF and lower levels of NT-proBNP (89.61 vs. 51.45, p = 0.04), but no other association was found with the recent use of any antiretroviral.

Multivariate analysis was performed, including levels of NT-proBNP as the dependent variable, with the independent variables being the glomerular filtration rate, triglyceride levels, RV10 (vascular risk) and the current use of 3TC, TDF and fAPV.

Higher levels of NT-proBNP were associated with the current use of fAPV (p < 0.005) and a lower glomerular filtration rate (p < 0.0005).

## Discussion

In our series of HIV patients aged over 40 years, an association was found between the current use of fosamprenavir and higher levels of NTproBNP and between lower levels of glomerular filtrate and higher levels of NTproBNP.

In both cases there may be a biologically plausible explanation. Lower levels of glomerular filtrate is a marker of vascular risk and, in many cases, an expression of subclinical organ injury.

In the French cohort [18], the recent use of fosamprenavir was associated with a higher incidence of acute myocardial infarction. There is increasing concern about cardiovascular morbidity and mortality in patients with HIV infection, and about this fact specific guides to risk management have been published and various studies were conducted to elucidate the role that some component of HAART may have in ischaemic events.

In that area, it seems a useful strategy to determine biomarkers such as NT-proBNP in subgroups of patients at particular risk (the elderly or those with classical cardiovascular risk factors) to evaluate the cardiovascular safety of antiretroviral drugs, in order to anticipate, as far as possible, the occurrence of ischaemic events.

The linear relationship of NT-proBNP with cardiovascular events could make it a good surrogate marker. High levels of NT-proBNP in HIV population have been associated with the presence of comorbidities in women.

In the study by Mansoor [16], NTproBNP levels in the general population were found to be associated with the presence of anaemia and renal dysfunction. In the case of women infected with HIV, no relation with the use of HAART was found. However, of the 454 infected women who participated in the study, only 60% were receiving HAART at the time of the analytical determination, compared to 89% in our study, this fact makes our study particularly suitable for detecting differences between those who receive antiretroviral treatment and those who do not. In the Spanish study by Serrano-Villar [19], an association was found between higher levels of NT-proBNP and increased intima-media thickness. In the present study, measurements were not conducted specifically to detect subclinical vascular disease, but studies such as the one mentioned advocate the use of NTproBNP as a surrogate marker of vascular disease. Duprez et al. [14] reported a higher risk of cardiovascular events among patients with HIV infection and higher levels of NT-proBNP than among those with lower levels. This relationship was controlled using traditional cardiovascular risk factors and other biomarkers such as highly sensitive C-reactive protein and D-dimer.

We acknowledge that our study is a cross-sectional one with a small sample size, and thus insufficient to draw firm conclusions; nevertheless, it is striking that even in this small series the factors associated with higher levels of NT-proBNP are those which could be linked to the occurrence of cardiovascular events.

## Conclusions

Even in this small serie, the factors associated with higher levels of NTproBNP are those which could be linked to the occurrence of cardiovascular events. This strongly suggests the usefulness of determining these levels, at least in subgroups at particular risk of such events.

According to our data higher levels of NT-proBNP were found among lower glomerular filtration rate and those using fAPV, on the other hand lower levels are in relation with the recent use of TDF.

Biomarkers such as NT-proBNP might be used in patients at moderate or high vascular risk in order to optimise the primary prevention of vascular events and perhaps even to optimise antiretroviral therapy.
